# Status Quo analysis of an exercise therapy care model in pediatric oncology during acute therapy: perspectives from patients, parents, siblings, and staff

**DOI:** 10.3389/fped.2026.1791439

**Published:** 2026-04-22

**Authors:** Lena Böhlke, Thorsten Simon, Barbara Hero, Dana Reinke, Amrei Friedrich, Nora Zoth, Freerk T. Baumann

**Affiliations:** 1 Department of Pediatric Hematology and Oncology, University Hospital of Cologne, Cologne, Germany; 2 Department I of Internal Medicine, Center for Integrated Oncology Cologne Bonn, University Hospital of Cologne, Cologne, Germany

**Keywords:** acute medical treatment, childhood cancer, exercise therapy, pediatric oncology, physical activity

## Abstract

**Background:**

In oncological pediatrics, exercise therapy is still not part of regular medical care in Germany and exercise therapy projects are currently financed by charity organizations. Nevertheless, the scientific evidence for the benefit of exercise therapy in pediatric oncology has led to the implementation of exercise therapy programs at most of the German hospitals. At the Pediatric Oncology Department of the University Hospital of Cologne the exercise therapy program was established in 2021. Between 2023 and 2025 a comprehensive survey was carried out to assess the perception of various groups such as patients, family and staff with offered program.

**Methods:**

A monocentric survey was conducted to assess the perception of patients, parents, siblings, and staff members regarding the exercise project using non-validated questionnaires. The questionnaires addressed the exercise project during intensive medical therapy in both the outpatient and inpatient phases. The questionnaires contained both open and closed questions and were evaluated using the following categories: “Communication/Education”, “Participation”, “Satisfaction”, “Need for Support”, “Online Exercise Sessions”, and “Barriers and Motives”.

**Results:**

The survey was completed by 33 patients, 63 parents, 14 siblings and 48 staff members. The evaluation of the survey showed that more than 50% of the patients and parents surveyed were satisfied with the existing program. Parents and staff have particularly noted the lack of exercise therapy programs for patients during their outpatient phases.

**Discussion:**

Despite the generally positive evaluation, patients, parents, siblings and staff still see potential for further development of the exercise therapy program, especially for the outpatient phases.

**Conclusion:**

Although the physical activity program is highly regarded, its potential is limited by gaps in outpatient care and inadequate sibling integration. To improve exercise therapy further, a holistic, family-centered approach must expand beyond the inpatient setting. Overcoming skepticism toward telehealth is crucial to ensuring accessible, sustainable exercise therapy outside the hospital.

**Clinical Trial Registration:** https://drks.de/search/de/trial/DRKS00034629/details, identifier (DRKS00034629).

## 1 Introduction

In Germany, approximately 2,250 children and adolescents between the ages of 0 and 18 years are diagnosed with cancer each year (1). There are about 60 acute care hospitals in Germany that offer cancer treatment for children and adolescents. These hospitals work together in the German Society for Pediatric Oncology and Hematology (GPOH). The complex interaction between the disease, the treatment and the corresponding side effects leads to a pronounced physical inactivity over a period of several months for the patients (2, 3). Although there is evidence in the literature that adjunctive exercise therapy can increase the activity levels of cancer patients and thus reduce decline in performance and other unwanted therapeutic burden (4, 5), physiotherapy is currently the only exercise-related intervention covered by German health insurers (6). Most of the existing early exercise therapy programs for children with cancer are not supported by health insurers and funded by charity organizations. Only three of the 60 pediatric oncology centers have selective contracts with individual health insurance companies that allow for partial funding of exercise therapy by the regular healthcare system (7).

Since January 2021 the Department of Pediatric Oncology at the University Hospital of Cologne, Germany has established an exercise therapy program completely funded by the Cologne parents organization and has become a member of the *Network ActiveOncoKids* (NAOK) (6). Our approach of exercise therapy for the Cologne site is based on existing concepts (7, 8) and recommendations of the current S2k guideline “Promotion of Exercise and Exercise Therapy in Pediatric Oncology” (9, 10).

Since a childhood cancer affects the entire family, a patient-centered approach involving other family members was chosen for establishing and implementing exercise therapy in pediatric oncology at the University Hospital of Cologne.

This survey aimed to evaluate the implementation and quality of exercise therapy, using the results to further improve our exercise therapy program. As this program is new, we also included members of the medical team in addition to patients, parents, and siblings in the survey. The results will be used to adapt the exercise therapy program to families’ preferences, identify obstacles to patient participation, and expand and improve the program.

## 2 Methods

### 2.1 Exercise therapy project

Since January 2021, the psychosocial team of the Department of Pediatric Oncology at the University Hospital of Cologne, Germany, has been expanded by an exercise therapy funded by the Cologne parents organization and has become a member of NAOK (6).

**Figure 1:**
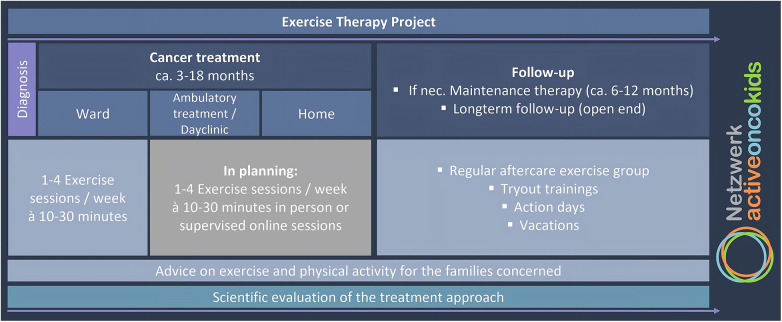
Structure of the exercise therapy project offered by the pediatric oncology department at university hospital, Cologne from the time of diagnosis, during cancer treatment and follow-up.

The concept of exercise therapy for the Cologne site is based on already established concepts and recommendations of the NAOK (7, 8). Both during cancer treatment and follow-up care, appropiate exercise therapy is offered, involving the whole family whenever possible (Figure 1). Patients aged two years and older receive 1–3 supervised exercise sessions per week of 10–30 min each during the inpatient phase from the time of diagnosis. Exercise sessions are supervised by an exercise scientist. They take place in patients' rooms or on the pediatric oncology ward. There is no separate room for exercise therapy. The content of the exercise therapy is always adapted to the age, wishes and needs of the patients. Exercise sessions incorporate a variety of elements, including endurance, strength, coordination, flexibility, sports games, and body awareness. The intensity of these exercises is adjusted to the patient's condition and ranges from low to high. Depending on the patient's condition, sessions may take place in or outside of bed. The sessions are designed with attention to potential disease- and therapy-related side effects, such as polyneuropathy. The risk of adverse events during exercise is minimized through individualized session design and supervised support, for example, checking medical history and blood values before an exercise session. When exercising with younger children, the focus is usually on playful activities. For example, strength training can be carried out in the form of an obstacle course. Older children, on the other hand, can do classic circuit training with weights. At present, exercise sessions were rarely held during the outpatient phase, both in the clinic, i.e., in the day clinics and outpatient clinic, and at the patient's home. In addition to exercise therapy, patients have the opportunity to exercise independently (e.g., playing table tennis, riding a toy Unimog or, playing table football).

### 2.2 Participants

Participants were enrolled in the program when they met the following criteria: (a) Patients aged 3–17 years with an oncologic disease, who had undergone anticancer therapy for at least 70 days and had been discharged home at least once, were included. Appropriate German language skills were required for participation. Patients who had completed their anticancer therapy more than 4 weeks previously were not included. In addition, the (b) parents/guardians of the participants, their (c) siblings aged 6 years and older and the (d) staff of the pediatric oncology department were surveyed.

### 2.3 Procedures

The study was designed as a single-arm, non-interventional, monocentric survey at Pediatric Hematology and Oncology at University Hospital Cologne, Germany. Ethical approval was granted by the Ethics Committee of Medical Faculty the University of Cologne (23-1127_1). Eligible participants were recruited between September 2023 and January 2025. A researcher identified eligible participants from clinic lists. Families were informed verbally and in writing that participation in the study was voluntary. Before participating in the study, legal guardians, patients, and siblings aged eight and older gave written consent for their data to be used anonymously for statistical analysis. Different versions of the consent form were used for those aged eight to 13 an those aged 14 to 17.

### 2.4 Data collection and development process

Based on previous research, specific non validated questionnaires were developed for following four target groups: (1) patients, (2) parents and guardians, (3) siblings, and (4) staff (Supplementary Material Files S5–S9). The patient and parent questionnaires are conceptually aligned to capture comparable perspectives on key program aspects. Questions for siblings and staff were adapted to reflect their roles and experiences with the program. During the development process, families from the target groups were involved in piloting the questionnaires. They were asked to complete the questionnaires and provide feedback on the clarity and comprehensibility of the questions. Based on their feedback, the questionnaires were revised and refined linguistically to improve their suitability and comprehensibility for the respective target groups. Ultimately, each population was asked to anonymously assess their perception of the current exercise program during acute anticancer therapy.

(1) The patient questionnaire was divided into five content areas: (a) general information about the exercise therapy program (4 items), (b) exercise therapy program on the ward (11 items), (c) exercise opportunities on the ward in addition to the existing exercise therapy program (5 items), (d) barriers and motivation to participate in exercise therapy (17/12 items), and (e) exercise therapy during the outpatient phases (9 items).
(2) The parents and guardians also received a questionnaire with four content topics: (a) general information about the exercise therapy program (5 items), (b) exercise therapy program on the ward (12 items), (c) exercise opportunities on the ward in addition to the existing of exercise therapy (6 items), (d) exercise therapy during outpatient phases (9 items).
(3) The patients’ siblings were asked about two topics in a questionnaire about exercise therapy program: (a) general information about exercise therapy (8 items), (b) barriers and motivation for joint exercise with the sibling (5/7).
(4) The pediatric oncology ward staff was surveyed on 15 items concerning the delivery of exercise therapy to patients during periods of acute medical therapy.

Depending on their age, children either completed the questionnaire independently or with assistance from a parent or caregiver. Participants aged 14 years and older were asked to complete the survey independently. For children younger than 14, parents or caregivers were instructed to provide support when needed. This assistance primarily involved reading the questions aloud, clarifying the wording in an age-appropriate manner, and recording the child's response, if necessary. Parents were asked to ensure that the answers reflected the child's views, not their own. Completing the questionnaires took between 30 and 45 min.

For the youngest participants, in particular, the procedure resembled a parent-assisted questionnaire rather than independent self-report. They survey consisted of short items with simple response options, using a 5-point (disagree—somewhat disagree—neutral—somewhat agree—agree) or 3-point Likert scale (patient age < 6 years; disagree—neutral—agree) was used to answer the questions (11). In addition, there was an option to enter wishes and comments as free text. These were presented sequentially to facilitate comprehension across different age groups.

### 2.5 Statistical analysis

The survey was analyzed using SPSS (Version 30.0) and Excel software. The survey results of patients and parents are presented comparatively in the categories “Communication/Education”, “Participation”, “Satisfaction”, “Need for Support” and “Online Exercise Sessions”. The survey results of siblings and staff are considered separately. To maintain focus on the research questions relevant to a broader clinical context, items specifically designed to evaluate internal institutional processes were excluded from the descriptive analyses.

## 3 Results

### 3.1 Participants

**Figure 2:**
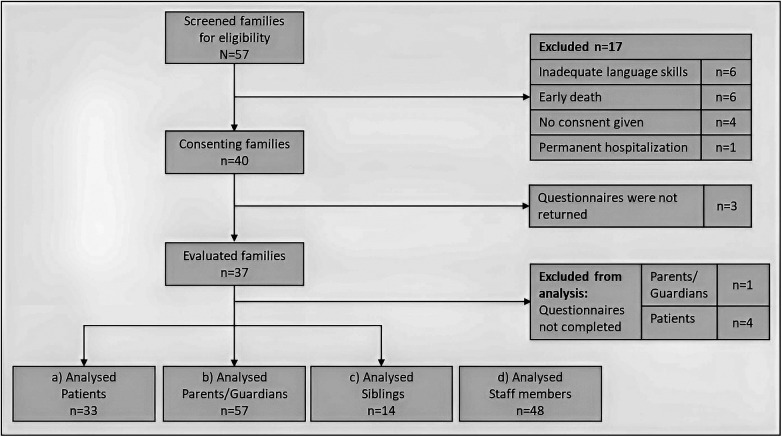
Illustration of participants flow reduction and data collection.

A total of 57 families were screened for the survey, 17 of which were excluded from participation due to inadequate language skills, early death, no consent given or permanent hospitalization. The family's willingness to participate in the survey amounted to 40, but ultimately only 37 families took part in the study. A total of 33 patients with different cancer entities, 63 parents or guardians, 14 siblings and 48 employees of the pediatric oncology department completed the questionnaires (Figure 2, Table 1).

**Table 1:** Main demographic and clinical characteristics of the participants; SD, standard deviation.

**Patients**	*n* = 33
Gender	m = 16, f = 17
Age (years), mean ± SD (range)	10.2 ± 4.9 (3–17)
Diagnosis	
Leukemia	*n* = 15 (45.5%)
Brain tumor	*n* = 2 (6%)
Sarcoma	*n* = 8 (24.2%)
Lymphoma	*n* = 3 (9%)
Neuroblastoma	*n* = 4 (12.1%)
Adenocarcinoma	*n* = 1 (3%)
**Parents/Guardians**	*n* = 63
Gender	m = 27, f = 36
Age (years), mean ± SD (range)	43.2 ± 6.9 (27–60)
**Siblings**	*n* = 14
Gender	m = 11, f = 3
Age (years), mean ± SD (range)	11.9 ± 4.8 (8–24)
**Staff**	*n* = 48
Function	
Nursing staff	*n* = 20 (41.7%)
Physician	*n* = 8 (16.6%)
Psychosocial care	*n* = 6 (12.5%)
Physical therapy	*n* = 5 (10.4%)
Others (a.o. secretarial staff, laboratory staff)	*n* = 9 (18.7%)

### 3.2 Survey of patients and parents/guardians

#### 3.2.1 Communication/education

**Figure 3:**
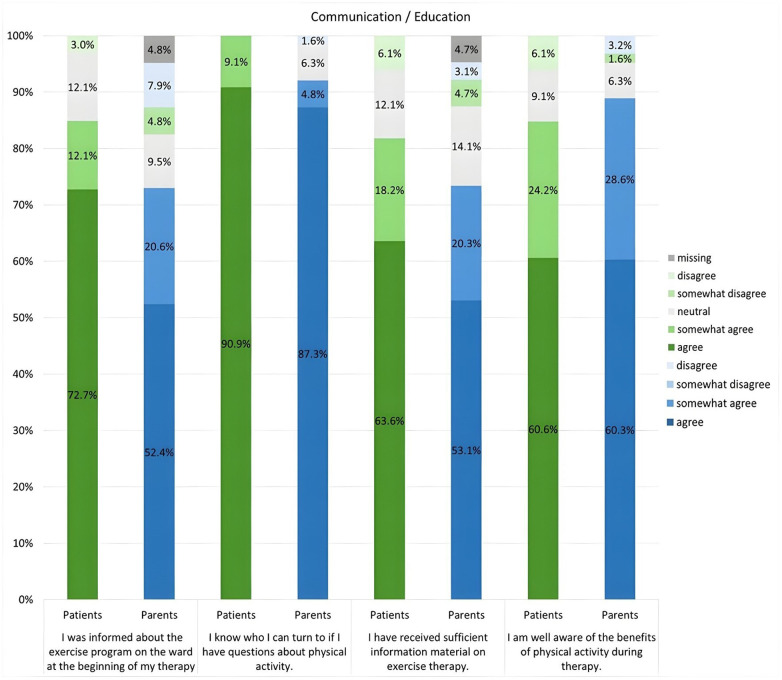
Survey results from patients and parents regarding “Communication and Education” in the context of the exercise therapy program.

The majority of patients (84.8%) and parents (73.0%) stated that they had been informed about the availability of an exercise therapy at the start of cancer treatment. They also knew who to contact with questions about physical activity during therapy (Figure 3).

#### 3.2.2 Participation/motivation

The majority of patients (75.4%) and parents (88.8%) reported that they or their children regularly participated in the exercise program during their inpatient stays. 91% of patients and 88.9% of parents agreed that the program helps them or their children to be active during their inpatient stays. 68.3% of parents strongly agreed and 15.9% somewhat agreed that they motivate their children to participate in exercise therapy.

The motives mentioned by patients for participating in the exercise program were “Improve muscles and endurance” (69.7%), “Coping with therapy” (69.7%), “Fun and joy” (69.7%) and “Distraction” (63.6%), “Body shaping” (54.5%) and “Because of others” (63.6%). The evaluation is available in Supplementary Material File S1. Patients most commonly cited the following obstacles to participating in exercise therapy: “I am suffering from the side effects of medical treatment” (42.5%), “I feel weak.” (30.3%) and “I am too tired and exhausted” (27.3%). The structural conditions of exercise therapy—i.e., organizational and program-related aspects such as how the program is offered, communicated, and presented—are not perceived as a barrier by the patients. Examples include “I am not asked if I want to participate in the exercise program” (3.0%). “The offer does not appeal to me”. (3.0%) and “I do not feel well informed about the exercise therapy program”. (6.1%). The evaluation is available in Supplementary Material File S2.

#### 3.2.3 Satisfaction

**Figure 4:**
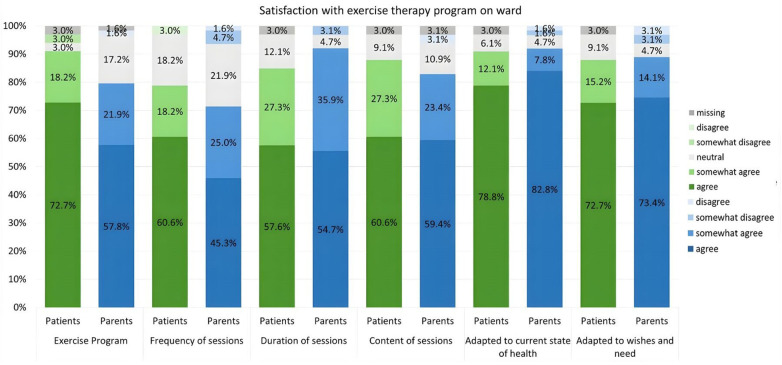
Survey results from patients and parents regarding “Satisfaction with exercise program on ward” in the context of the exercise therapy program.

Regarding general satisfaction with the program, 72.7% of patients and 57.8% of parents selected the highest category (“agree”). Both patients and parents are also very satisfied with the general conditions of the exercise sessions in the inpatient setting (frequency, duration, content, etc.) (Figure 4). 90.9% of patients and 86.0% of parents reported having fun during the exercise sessions. Furthermore, 45.5% of patients and 34.4% of parents strongly agree that they are satisfied with exercise opportunities outside of exercise therapy sessions on the ward.

#### 3.2.4 Need for support

**Figure 5:**
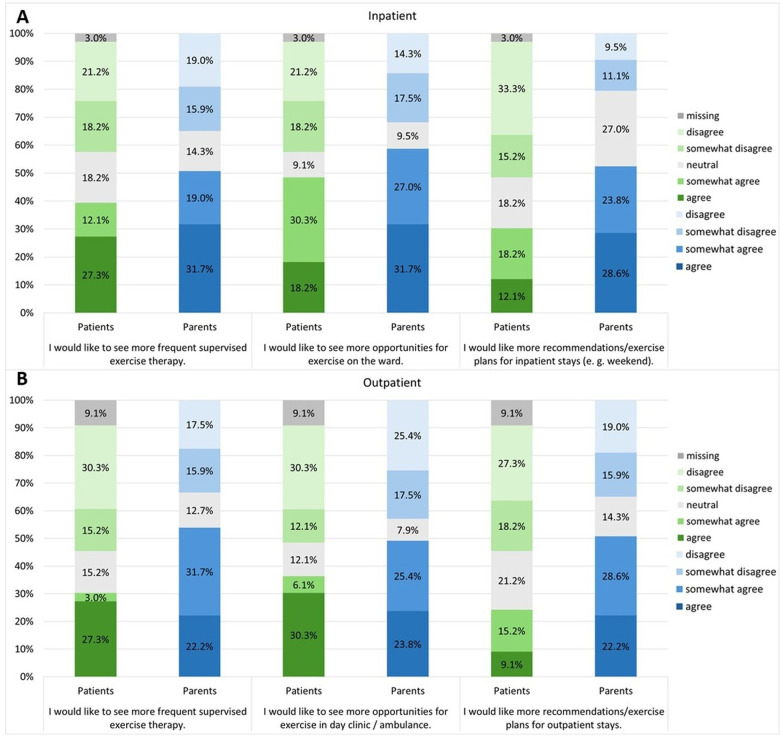
Survey results from patients and parents regarding “Need for support” in the context of the exercise therapy program during **(A)** inpatient and **(B)** outpatient stays.

More than 50% of the parents completely agree or somewhat agree that they would like to have more recommendations or training plans for their children during both inpatient and outpatient stays. 52.4% of parents agree or somewhat agree that they would like to have more contact with an exercise therapist during the outpatient phases. 50.8% of parents completely or somewhat agree that they would like their children to have their own individual exercise program during their stay in hospital (Figure 5). Responses to the free text fields show that patients and parents would like to have an exercise room and additional opportunities for unsupervised exercise on the ward, especially for young children. They also expressed a need for exercise opportunities at weekends and in the day clinic/outpatient clinic.

#### 3.2.5 Online exercise sessions

73.9% of the patients and 87.3% of the parents surveyed agree that they are technically equipped to participate in supervised online training sessions from home. In addition, 60.8% of patients and 69.8% of parents agree or partially agree that they have time to participate. 8.7% of the patients can imagine taking part in online training sessions. However, 42.8% of the parents could imagine their children participating in such sessions. While 50.8% of the patients surveyed could not imagine that online training sessions could be fun, 39.6% of the parents surveyed could imagine that their children would enjoy this form of training. The Figure is available in Supplementary Material File S3.

### 3.3 Survey of siblings

64.3% of the siblings attend elementary school, 21.4% attend secondary school and 14.2% are in vocational training or higher education. 92.8% of the siblings reported that they had not been informed about exercise therapy program. In addition, 57.2% of siblings reported that they did not know who to contact on the oncology children's ward if they had questions about sport and exercise therapy. Half of the siblings surveyed responded that they would like to take part in their siblings' exercise sessions more often. The main reasons for doing sport and exercise with their sibling were “Fun and joy” (78.6%), “Spend time together” (71.4%) and “Forget about the illness for a while” (57.2%). 85.7% of the siblings stated that they were not afraid of moving around with their sick sibling. Half of the siblings stated that they would prefer to do something else. For further information see Supplementary Material File 4.

### 3.4 Survey of staff

The staff members are divided into the following professions: Nursing (41.7%), doctors (16.7%), physiotherapy (10.4%), psychosocial care (12.5%) and other (18.8%). 35.4% of the staff members have been working at pediatric oncology department for more than ten years, 29.2% for between five and ten years, 27.1% for between one and five years and 8.3% for less than one year.

**Figure 6:**
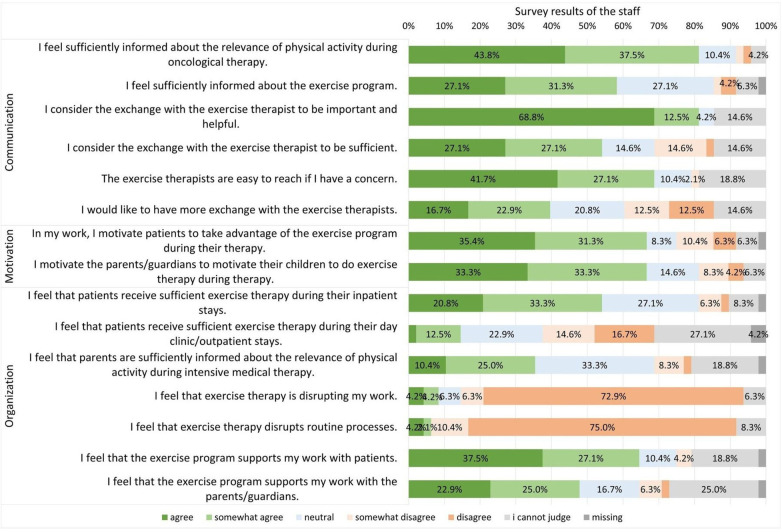
Survey results from staff in context of the exercise therapy program.

The majority of staff feel well informed about the relevance of physical activity during medical therapy (81.3%) and about the exercise program (58.4%). Only 14.6% of staff are completely or somewhat of the opinion that patients receive sufficient exercise therapy in the day clinic/outpatient clinic. This is reflected in the free comments such as: “In my opinion, the exercise program must also be offered in the day clinic” and “Another exercise therapist to cover the largest possible group of patients on several days of the week”. 8.4% of the staff surveyed stated that their actual work or routine processes (6.3%) were disrupted by the exercise program (Figure 6).

## 4 Discussion

Based on the fact that exercise programs can be carried out safely during intense medical therapy and have a positive effect on physical and mental health (12–14). Evaluating the perception of a newly established exercise program in a clinical setting—modeled after the German network ActiveOncoKids—offers the advantage of identifying organizational, communicative, and functional difficulties at an early stage. Since the existing exercise therapy program was not finally set up at the time of evaluation, adjustments could be easily made based on the survey results. The primary objective is to improve the quality and efficiency of exercise therapy for children with cancer undergoing anticancer treatment.

The evaluation results demonstrate high levels of satisfaction with the current exercise program (see 2.1). The individual adaptation of sessions to the patients' health status and specific needs was highlighted as a particularly positive aspect. This patient-centered approach aligns with the recommendations of the S2K guideline (9) and the ActiveOncoKids network (10), which form the structural foundation of the program. Despite the families' general satisfaction with the frequency of therapeutically supervised sessions, approximately half of the parents expressed a desire for a further increase in the offerings. This highlights a discrepancy between the achieved activity levels and current physical activity recommendations. While German guidelines prescribe at least 180 min of daily activity for children aged four to six, and at least 90 min of moderate-to-vigorous activity for those aged six to 17 (15), pediatric oncology patients rarely meet these targets in everyday clinical practice (2, 16). Although the exercise program provides incentives to follow these recommendations, the program alone cannot bridge this gap.

The presented results demonstrate high acceptance of the inpatient exercise program. A large majority of patients and parents confirmed its effectiveness in promoting physical activity. This positive perception is supported by strong intrinsic motivational factors (17–19) among patients, such as improving strength and endurance and actively coping with therapy. However, the analysis reveals a “motivation gap” among the treatment team: While 68.3% of parents motivate their children to participate, only 35.4% of healthcare professionals do. Furthermore, only one-third of staff (33.3%) encourage parents to take on a motivating role. This discrepancy indicates that the potential of parents as an important resource is not currently being maximized. Parents understand their children's behavior. They are important in getting their kids to exercise at home. In line with Keogh et al. (20), our data underscores the necessity of strengthened interprofessional collaboration between exercise scientists, nursing staff, and physicians. Optimized communication within the multidisciplinary team could empower medical personnel to address families more effectively and strengthen social support, which is a key factor for therapy adherence (21, 22).

As the exercise programs are not part of standard medical care, but are financed by grants, there are often limited facilities and/or staff (6). The identification of organizational barriers provides a clinically relevant explanation for this: limited personnel capacity and infrastructural constraints restrict the expansion of the supervised program (17, 23, 24). For instance, the lack of a dedicated exercise room means that therapy sessions must often take place in hallways or patient rooms, which reduces therapeutic variety. Furthermore, the offerings largely cease during outpatient phases due to a lack of established structures. The present data suggests that unsupervised exercise options on the ward are perceived as fully sufficient by only a minority of respondents. The explicit desire of parents for additional training plans and suggestions for weekends and holidays underscores the need for low-threshold resources for self-organization. Consequently, further development of the inpatient infrastructure—particularly the creation of a child-friendly exercise room and the provision of information materials for home use—is a central recommendation to sustainably close the gap between clinical guidelines and daily reality.

There is further room for improvement regarding the involvement of the broader family environment. Since 35.7% of siblings reported receiving little to no support from the program, future strategies should aim to systematically integrate siblings into exercise therapy. Research findings indicate that siblings frequently exhibit post-traumatic stress symptoms and often report a reduced quality of life in various domains, including emotional, familial, and social aspects (25, 26). It is therefore of great relevance to improve the integration of siblings into exercise therapy in the future. In Addition, siblings can promote physical activity by providing social interaction and combating loneliness (19, 27). That is why, creating a holistic “supportive environment” involving all stakeholder groups must be a central goal of future optimizations to enhance the quality of the exercise program sustainably.

It has already been shown that the exercise behavior of parents of children with cancer also decreases during therapy, especially during inpatient stays (28). Parental exercise behavior can have an influence on the child's behavior during and after cancer therapy (29). In view of this, exercise programs for parents during their children's cancer therapy should be considered, which is supported by our survey. This intervention has the potential to make a contribution to reducing family inactivity while promoting activity.

As part of the conceptual considerations regarding the implementation of online exercise sessions in the outpatient phases, items were used to record the perspectives of patients and parents. The survey highlights a discrepancy between technical feasibility and actual acceptance: while most patients have the necessary equipment (laptop, tablet) and time, 65.2% of the patients who are older than six years cannot imagine participating in a telemedicine exercise session. Despite greater openness among parents, the data shows an unexpected lack of enthusiasm. Since the present study did not detail the specific organization of these sessions, the reasons for this hesitation remain unclear. This finding is particularly relevant as international studies have already demonstrated that telemedicine-based programs to increase physical activity are both feasible and effective within this target group (30, 31). High adherence rates in these studies further suggest significant potential. The use of telemedicine offers the crucial advantage of continuing exercise sessions during the outpatient phases of treatment. Through this digital expansion, the overall frequency of sessions could be increased and geographical barriers could be overcome (32). The insights gained here thus serve as a sound basis for the design of future intervention studies to further evaluate the actual benefits of online sessions in pediatric oncology.

### 4.1 Limitations and strengths

This study has several limitations that affect the interpretation and generalizability of its results. While the participation of 37 families yielded meaningful findings, the subgroup size, especially among siblings (*n* = 14), was small. This limits the validity and reliability of subgroup-specific conclusions. Additionally, the results are not easily transferable because the study was conducted in a pediatric oncology department at a single center in Germany. Structural and institutional factors may differ in other institutions.

The study surveyed children across a wide age range (3–17 years). The questionnaire was completed by younger participants, especially preschool-aged children, with the help of a parent or caregiver. The caregiver read the questions aloud and recorded the child's responses. While this approach enabled the inclusion of very young children, their responses may have been influenced by parental interpretation or assistance. Additionally, young children's ability to comprehend certain concepts may be limited. Caregiver involvement also raises the possibility that caregiver perspectives may have been indirectly represented more than once within the dataset. Therefore, the results from the youngest participants should be interpreted with caution. Future studies could benefit from using age-appropriate instruments or interview-based approaches designed specifically for very young children.

The study was subject to potential selection bias of committed families who were motivated to take part in the underlying survey. Consequently, vulnerable or less committed families may have been underrepresented. Additionally, the data collection relied on subjective self-reporting using self-constructed questionnaires. This could lead to socially desirable responses that overstated the satisfaction or perceived benefits of the program.

The cross-sectional design only captured a single point in time, so it did not reflect changes in perception or participation throughout the treatment process (e.g., during relapse or transition to aftercare). Furthermore, the telehealth study lacked depth because it did not use qualitative methods, such as interviews or focus groups, to better understand the barriers and contextual factors influencing telehealth uptake.

Nevertheless, the study has several strengths that highlight its relevance and contribution to the development of pediatric oncology. It takes into account the needs of patients, families and medical staff, thus incorporating multiple perspectives and enabling a comprehensive evaluation and optimization of the programs. The study showed a remarkably high level of interest: 37 of the 57 families screened took part, underlining the importance of the topic for those affected. The results have direct implications for the development of exercise programs in pediatric oncology. Although the results are primarily tailored to the exercise program at the University Hospital of Cologne, they provide valuable insights for the implementation of similar programs in other clinics.

Additionally, the study offers clear starting points for optimization, such as greater sibling involvement in the program and enhanced cooperation between medical staff and exercise therapists. By focusing on the S2k guideline, “Promotion of Exercise and Exercise Therapy in Pediatric Oncology”, the results are embedded in the context of established quality standards, further strengthening their significance and practical relevance. Another advantage of the study is the survey of the technical requirements for telemedicine services. Most participants have the necessary equipment, creating a solid basis for developing digital health services. Overall, the study provides valuable starting points for developing innovative, patient-centered care concepts in pediatric oncology.

## 5 Conclusion

The study conducted in the Department of Pediatric Oncology at the University Hospital of Cologne underlines the importance and popularity of physical activity programs during intensive medical therapy for pediatric patients, their families and staff. The study found that patients and families respond positively to these programs, especially during their inpatient stay. At the same time, the study identified areas requiring particular attention in the project's further development. (1) There are deficits in outpatient and day clinic care for patients regarding exercise therapy. This underscores the importance of extending exercise programs beyond the inpatient setting. (2) The present approach focuses on the family of a pediatric patient. A significant finding is that siblings do not feel sufficiently integrated into exercise therapy. Encouraging sibling participation in exercise therapy could support the entire family with regard to childcare and physical activity. (3) The observed reluctance to adopt telehealth formats underscores the importance of thoughtful design and effective communication when developing digital physical activity programs. Conducting interviews could provide deeper insights into the contextual factors that influence the adoption of telehealth. This would support the development of user-centered, accessible telehealth approaches. (4) Improving communication strategies between exercise therapists and staff, particularly by involving more healthcare professionals, could increase program effectiveness and accessibility.

## Data Availability

The raw data supporting the conclusions of this article will be made available by the authors, without undue reservation.

## References

[1] Spix C Erdmann F Grabow D Ronckers C. Childhood and adolescent cancer in Germany—an overview. J Health Monit. (2023) 8(2):79–94. 10.25646/11438 37408714 PMC10318562

[2] Götte M Kesting S Winter C Rosenbaum D Boos J. Comparison of self-reported physical activity in children and adolescents before and during cancer treatment. Pediatr Blood Cancer. (2014) 61(6):1023–8. 10.1002/pbc.24898 24357259

[3] Winter C Müller C Hoffmann C Boos J Rosenbaum D. Physical activity and childhood cancer. Pediatr Blood Cancer. (2010) 54(4):501–10. 10.1002/pbc.22271 19743298

[4] Wurz A McLaughlin E Lategan C Ellis K Culos-Reed SN. Synthesizing the literature on physical activity among children and adolescents affected by cancer: evidence for the international pediatric oncology exercise guidelines (iPOEG). Transl Behav Med. (2021) 11(3):699–708. 10.1093/tbm/ibaa136 33538309 PMC8033595

[5] Braam KI van der Torre P Takken T Veening MA van Dulmen-den Broeder E Kaspers GJL. Physical exercise training interventions for children and young adults during and after treatment for childhood cancer. Cochrane Database Syst Rev. (2016) 3:CD008796. 10.1002/14651858.CD008796.pub3 27030386 PMC6464400

[6] Götte M Söntgerath R Gauß G Wiskemann J Buždon M Kesting S. A national implementation approach for exercise as usual care in pediatric and adolescent oncology: network ActiveOncoKids. Pediatr Exerc Sci. (2022) 34(4):219–26. 10.1123/pes.2021-0218 35700978

[7] Söntgerath R Küpper L Wulftange M Schepper F Christiansen H. Bewegungsförderung in der pädiatrischen onkologie—strukturelle voraussetzungen und finanzierungsmöglichkeiten anhand des leipziger bewegungskonzepts. Klin Padiatr. (2019) 231(3):150–6. 10.1055/a-0856-7495 30934088

[8] Kesting S Götte M Seidel C Müller C Michel T Krüger M Bewegungs- und sportförderung in der pädiatrischen onkologie am universitätsklinikum münster—erfahrungen und ergebnisse aus 5 jahren. B&G Bewegungstherapie Gesundheitssport. (2016) 32(02):60–5. 10.1055/s-0042-103432

[9] Gauß G Kesting S Creutzig U Baumann F Boos J Dirksen U Neue AWMF-S2k-leitlinie bewegungsförderung und bewegungstherapie in der pädiatrischen onkologie. Monatsschr Kinderheilkd. (2022) 170(6):548–50. 10.1007/s00112-022-01474-z

[10] Götte M Gauß G Dirksen U Driever PH Basu O Baumann FT Multidisciplinary network ActiveOncoKids guidelines for providing movement and exercise in pediatric oncology: consensus-based recommendations. Pediatr Blood Cancer. (2022) 69(11):e29953. 10.1002/pbc.29953 36073842

[11] Ferrando PJ Morales-Vives F Casas JM Muñiz J. Likert scales: a practical guide to design, construction and use. Psicothema. (2025) 37(4):1–15. 10.70478/psicothema.2025.37.24 41187005

[12] Yildiz Kabak V Calders P Duger T Mohammed J van Breda E. Short and long-term impairments of cardiopulmonary fitness level in previous childhood cancer cases: a systematic review. Support Care Cancer. (2019) 27(1):69–86. 10.1007/s00520-018-4483-8 30251066

[13] Söntgerath R Eckert K. Impairments of lower extremity muscle strength and balance in childhood cancer patients and survivors: a systematic review. Pediatr Hematol Oncol. (2015) 32(8):585–612. 10.3109/08880018.2015.1079756 26558954

[14] Götte M Kesting SV Winter CC Rosenbaum D Boos J. Motor performance in children and adolescents with cancer at the end of acute treatment phase. Eur J Pediatr. (2015) 174(6):791–9. 10.1007/s00431-014-2460-x 25428233

[15] Pfeifer K Rütten A. Nationale empfehlungen für bewegung und bewegungsförderung. Das Gesundheitswesen. (2017) 79(S 01):S2–3. 10.1055/s-0042-123346 28399579

[16] Götte M Basteck S Beller R Gauß G Schmidt S Burchartz A Physical activity in 9–15 year-old pediatric cancer survivors compared to a nationwide sample. J Cancer Res Clin Oncol. (2022) 149(8):4719–29. 10.1007/s00432-022-04392-5 36224439 PMC10349711

[17] Götte M Kesting S Winter C Rosenbaum D Boos J. Experience of barriers and motivations for physical activities and exercise during treatment of pediatric patients with cancer. Pediatr Blood Cancer. (2014) 61(9):1632–7. 10.1002/pbc.25071 24753116

[18] Grimshaw SL Taylor NF Mechinaud F Conyers R Shields N. Physical activity for children undergoing acute cancer treatment: a qualitative study of parental perspectives. Pediatr Blood Cancer. (2020) 67(6):e28264. 10.1002/pbc.28264 32277806

[19] Petersen NN Larsen HB Pouplier A Schmidt-Andersen P Thorsteinsson T Schmiegelow K Childhood cancer survivors’ and their parents’ experiences with participation in a physical and social intervention during cancer treatment: a RESPECT study. J Adv Nurs. (2022) 78(11):3806–16. 10.1111/jan.15381 35942568 PMC9804908

[20] Keogh JWL Pühringer P Olsen A Sargeant S Jones LM Climstein M. Physical activity promotion, beliefs, and barriers among Australasian oncology nurses. Oncol Nurs Forum. (2017) 44(2):235–45. 10.1188/17.ONF.235-245 28222085

[21] Adamovich T Watson R Murdoch S Giovino L Kulkarni S Luchak M Barriers and facilitators to physical activity participation for child, adolescent, and young adult cancer survivors: a systematic review. J Cancer Surviv. (2024) 18(2):245–62. 10.1007/s11764-022-01217-9 35665472

[22] El-Ayadi M Druivenga K Perwein T Nussbaumer G Spreafico F Massimino M General support of physical exercise programs in pediatric oncology but differences in perception by childhood cancer care professionals at European and North-African/Arab centers. EJC Paediatr Oncol. (2024) 3:100147. 10.1016/j.ejcped.2024.100147

[23] Ross WL Le A Zheng DJ Mitchell H-R Rotatori J Li F Physical activity barriers, preferences, and beliefs in childhood cancer patients. Support Care Cancer. (2018) 26(7):2177–84. 10.1007/s00520-017-4041-9 29383508

[24] Mizrahi D Wakefield CE Simar D Ha L McBride J Field P Barriers and enablers to physical activity and aerobic fitness deficits among childhood cancer survivors. Pediatr Blood Cancer. (2020) 67(7):e28339. 10.1002/pbc.28339 32386117

[25] Alderfer MA Long KA Lown EA Marsland AL Ostrowski NL Hock JM Psychosocial adjustment of siblings of children with cancer: a systematic review. Psychooncology. (2010) 19(8):789–805. 10.1002/pon.1638 19862680

[26] Long KA Lehmann V Gerhardt CA Carpenter AL Marsland AL Alderfer MA. Psychosocial functioning and risk factors among siblings of children with cancer: an updated systematic review. Psychooncology. (2018) 27(6):1467–79. 10.1002/pon.4669 29441699

[27] Schmidt-Andersen P Fridh MK Müller KG Anna P Hjalgrim LL Faigenbaum AD Integrative neuromuscular training in adolescents and children treated for cancer (INTERACT): study protocol for a multicenter, two-arm parallel-group randomized controlled superiority trial. Front Pediatr. (2022) 10:833850. 10.3389/fped.2022.833850 35359909 PMC8964065

[28] Ohnmacht C Niemeyer CM Pahl A Gollhofer A Puzik A. Assessing the physical activity of parents of children suffering from cancer: a cross-sectional study (2024). 10.1186/s12889-025-25455-5 PMC12621409 41250056

[29] Schmidt-Andersen P Boensvang NN Pouplier A Lykkedegn S Hasle H Müller K Exploring motivation for engaging in exercise during the first six months of childhood cancer treatment: a qualitative study. Eur J Cancer Care. (2025) 2025(1):14. 10.1155/ecc/8305173

[30] Peli L Inselvini E Cogliati M Finazzi E Gorio C Angeloni A Telemedicine-based adapted physical activity programs for pediatric oncology patients in active oncological care: a feasibility study. Front Oncol. (2025) 15:1634626. 10.3389/fonc.2025.1634626 40989703 PMC12450653

[31] Lambert G Alos N Bernier P Laverdière C Kairy D Drummond K Home-based telehealth exercise intervention in early-on survivors of childhood acute lymphoblastic leukemia: feasibility study. JMIR Cancer. (2021) 7(2):e25569. 10.2196/25569 34132645 PMC8277387

[32] Anawade PA Sharma D Gahane S. A comprehensive review on exploring the impact of telemedicine on healthcare accessibility. Cureus. (2024) 16(3):e55996. 10.7759/cureus.55996 38618307 PMC11009553

