# Supplementary material for: Status Quo analysis of an exercise therapy care model in pediatric oncology during acute therapy: perspectives from patients, parents, siblings, and staff
Source: Front Pediatr. 2026 Apr 22;14:1791439. doi: 10.3389/fped.2026.1791439 (PMC13144044; doi:10.3389/fped.2026.1791439)
Supplement: Supplementary file 1 [file Datasheet1.pdf]

Translated Version

Patients under 6 years of age – Acute Therapy

Contact

Exercise Scientist
Paediatric Oncology and Haematology
Phone: +49 221 478-42646
E-mail: lena.boehlke@uk-koeln.de

Dear___________________________________

As part of the

Status Quo Analysis of the Exercise Project
within the Department of Paediatric Oncology at University Hospital Cologne

we are conducting a survey on the provision of sports and exercise therapy services in pediatric oncology at University Hospital Cologne. Since December 2020, exercise therapy has been offered in addition to the existing treatment services. The purpose of this survey is to identify potential barriers that may limit access to exercise therapy. Our goal is to sustainably improve the structure of exercise therapy and to adapt it to the individual needs and preferences of patients.

Instructions for completing the questionnaire:

- If you are still too young to complete it by yourself, your parents may of course help you.
- There are no "right" or "wrong" answers.
- If a question does not really apply to you, or if it is difficult for you to decide, please choose the answer that feels most right to you.
- Please mark the answer that applies to you with an "X."

Thank you very much for your participation – your help means a lot to us!

Date			.			.				

ID										
Date			.			.				

Who fills out the questionnaire?

☐ Child / adolescent alone
☐ Child / adolescent with parent / caregiver
☐ Parent alone

You have been in oncological treatment for some time now and have become familiar with the **exercise offerings**. We will now ask you a few questions about the offer during your **inpatient stays.**

General questions about exercise therapy program	Agree	neutral	Disagree
1. At the beginning of my therapy, I was informed about the range of exercise on the ward.	☐	☐	☐
2. I know who to contact if I have questions about exercise and/or sport.	☐	☐	☐
3. I have received a sufficient amount of information material on exercise therapy.	☐	☐	☐
4. I know well about the importance of physical activity during therapy.	☐	☐	☐

Questions about sports and exercise therapy on the ward	Agree	neutral	Disagree
5. I regularly take advantage of the exercise offer on the ward.	☐	☐	☐
6. The exercise offer helps me to be active.	☐	☐	☐
7. I am satisfied with the exercise program.	☐	☐	☐
8. I enjoy the exercise sessions.	☐	☐	☐
9. The exercise sessions are adapted to my current state of health.	☐	☐	☐
10. The exercise sessions are adapted to my wishes and needs.	☐	☐	☐
11. I am satisfied with the frequency of exercise sessions.	☐	☐	☐
12. I would like to see more exercise therapy offers during inpatient stays.	☐	☐	☐
13. I am satisfied with the duration / length of the exercise sessions.	☐	☐	☐
14. The content of the exercise sessions is varied.	☐	☐	☐

ID										
Date			.			.				

15. I would like to see more recommendations/training plans/ideas to move outside of exercise therapy (e.g. on weekends).	☐	☐	☐

There is also the possibility to move around on the ward outside of exercise therapy. We would now like to find out whether you are satisfied with the exercise options on the ward.

**Questions about movement options on the ward**			
	Agree	neutral	Disagree
16. I am satisfied with the exercise possibilities on the ward (vehicle fleet, table tennis, table football).	☐	☐	☐
17. I would like to see more opportunities to move around on the ward.	☐	☐	☐
18. It would be easier for me to move around the ward if there was more material for it.	☐	☐	☐
19. I would like to move more on the ward, but I don't know how.	☐	☐	☐

20. We would like to know if there is anything we can improve in the movement therapy offer **on the ward**. Suggestions are welcomed here:

____

____

____

____

____

____

____

____

ID										
Date			.			.				

Now we would like to learn more about what obstacles there are for you to take advantage of the exercise program on the ward.

**Subjective barriers to participation in exercise therapy**			
	Totally true	Partly / partly	Not Applicable
21. I feel too tired and exhausted.	☐	☐	☐
22. I suffer from side effects of medical therapy (nausea, vomiting, etc.).	☐	☐	☐
23. I feel weak.	☐	☐	☐
24. I have problems moving (dizziness, balance).	☐	☐	☐
25. I feel low on energy.	☐	☐	☐
26. I have no motivation.	☐	☐	☐
27. I'm in a bad mood.	☐	☐	☐
28. I prefer to stay in bed and occupy myself differ-ently.	☐	☐	☐
29. I'm afraid of too much exertion and injuries.	☐	☐	☐
30. I'm afraid of doing something wrong.	☐	☐	☐
31. I didn't like to move before the diagnosis.	☐	☐	☐
32. I only move because others want me to.	☐	☐	☐
33. The offer does not appeal to me.	☐	☐	☐
34. I am not asked whether I would like to take ad-vantage of the exercise offer.	☐	☐	☐
35. I don't feel well informed about exercise ther-apy.	☐	☐	☐
36. I am often on the ward on weekends, which is why I miss the exercise offer on the ward.	☐	☐	☐
37. I'm rarely on the ward.	☐	☐	☐

ID										
Date			.			.				

Now we would like to learn more about the reasons why you participate in exercise therapy.

Subjective motives for participating in exercise therapy			
	Totally true ☺	Partly / partly 😐	Not Applicable ☹
I take advantage of the exercise program, ...			
38. ... to strengthen/maintain my muscles and endurance.	☐	☐	☐
39. ... to remain self-employed.	☐	☐	☐
40. ... in order to be able to keep up with the other children and adolescents after acute therapy.	☐	☐	☐
41. ... to get through the therapy well.	☐	☐	☐
42. ... to sleep better.	☐	☐	☐
43. ... to shape my body.	☐	☐	☐
44. ... to pass the time.	☐	☐	☐
45. ... to improve my mood.	☐	☐	☐
46. ... to distract me.	☐	☐	☐
47. ... to feel "normal".	☐	☐	☐
48. ... to have fun and joy.	☐	☐	☐
49. ... Because others want me to move.	☐	☐	☐

ID									
Date			.			.			

Between hospital stays, you always spend some time at home. During this time, you usually have little contact with an exercise therapist. We would now like to ask you a few questions about the exercise offer during **ambulant / outpatient stays**.

**Questions about exercise during outpatient phases**			
	Totally true	Partly / partly	Not Applicable
	☺	😐	☹
50. During my outpatient phases, I would like to have regular contact (in person/by phone) with an exercise therapist.	☐	☐	☐
51. I would like to have more opportunities to exercise during my outpatient stays in the day-clinic / ambulatory.	☐	☐	☐
52. I would like to see more recommendations/training plans for the outpatient phases that motivate me to exercise.	☐	☐	☐
53. I would like to see more supervised exercise therapy offers during my outpatient stays.	☐	☐	☐

54. What could an exercise program look like that you would participate in during your outpatient phases? Suggestions are welcomed here:

____

____

____

____

____

____

____

____

Thank you very much! ☺
