# Supplementary material for: Status Quo analysis of an exercise therapy care model in pediatric oncology during acute therapy: perspectives from patients, parents, siblings, and staff
Source: Front Pediatr. 2026 Apr 22;14:1791439. doi: 10.3389/fped.2026.1791439 (PMC13144044; doi:10.3389/fped.2026.1791439)
Supplement: Supplementary file 3 [file Datasheet3.pdf]

Translated Version

Parents and Legal Guardians – Acute Therapy

Contact

Exercise Scientist
Paediatric Oncology and Haematology
Phone: +49 221 478-42646
E-Mail: lena.boehlke@uk-koeln.de

As part of the

Status Quo Analysis of the Exercise Project within the Department of Pediatric Oncology at University Hospital Cologne

we are conducting a survey on the provision of sports and exercise therapy services in pediatric oncology at University Hospital Cologne. Since December 2020, exercise therapy has been offered in addition to the existing treatment services. The purpose of this survey is to identify potential barriers that may limit access to exercise therapy. Our goal is to sustainably improve the structure of exercise therapy and to adapt it to the individual needs and preferences of patients.

Notes on completing the questionnaire:

- If a question applies to you less or you find it difficult to choose an answer, please tick the one that most spontaneously fits you.
- Please mark the appropriate answer with an “X.”
- Places where we ask you to write something down are indicated by a box.

Thank you very much for taking the time!

Date			.			.				

ID										
Date			.			.				

Demographic Information

Last Name	
First Name	
Postal Code, City/Town	
Date of Birth	___________.____________<br>Month Year
Place of Birth	
Nationality	
Gender	☐ female ☐ male ☐ diverse
Marital Status	☐ married ☐ single<br>☐ divorced ☐ single Parent<br>☐ separated
Highest Level of Education	☐ No Degree<br>☐ Primary School<br>☐ Lower Secondary School<br>☐ Intermediate Secondary School<br>☐ Secondary School Diploma<br>☐ University of Applied Sciences<br>☐ University Degree
Occupation	
Number of Children	☐ 0 ☐ 2 ☐ 4<br>☐ 1 ☐ 3 ☐ more than 4
Travel Distance to the Clinic	___________ kilometers ___________ minutes
How long ago was your child's diagnosis?	☐ between 2 und 6 months<br>☐ more than 6 months
Child's Age	______ Years
Child's Diagnosis	

ID										
Date			.			.				

Your child has now been undergoing oncology treatment for some time and has become familiar with the **exercise program**. We would like to ask you a few questions about the program during your child's **inpatient stays**.

**General Questions About Sports and Exercise Therapy**					
	Agree	Some-what agree	Neutral	Some-what disa-gree	Disa-gree
1. I was informed about the exercise program at the beginning of the therapy.	☐	☐	☐	☐	☐
2. I know who to contact if I have questions about exercise and/or sports.	☐	☐	☐	☐	☐
3. I have received sufficient information materi-alstable about exercise therapy.	☐	☐	☐	☐	☐
4. I am well-informed about the importance of phys-ical activity during the therapy.	☐	☐	☐	☐	☐
5. I would like more information on the topic of physical activity during my child's oncology treat-ment.	☐	☐	☐	☐	☐
**Questions About Inpatient Exercise Therapy**					
	Agree	Some-what agree	Neutral	Some-what disa-gree	Disa-gree
6. During inpatient stays, my child is regularly of-fered exercise therapy.	☐	☐	☐	☐	☐
7. I encourage my child to participate in the exercise therapy sessions during inpatient stays.	☐	☐	☐	☐	☐
8. The exercise program helps my child stay active while on the ward.	☐	☐	☐	☐	☐
9. I am satisfied with the exercise program for my child.	☐	☐	☐	☐	☐
10. My child enjoys the exercise therapy sessions.	☐	☐	☐	☐	☐
11. The exercise therapy sessions are adapted to my child's current health condition.	☐	☐	☐	☐	☐
12. The exercise program is tailored to my child's needs and preferences.	☐	☐	☐	☐	☐
13. I am satisfied with the frequency of exercise ses-sions.	☐	☐	☐	☐	☐
14. I would like my child to have exercise therapy ses-sions more frequently during inpatient stays.	☐	☐	☐	☐	☐
15. I am satisfied with the duration/length of the ex-ercise therapy sessions.	☐	☐	☐	☐	☐

ID										
Date			.			.				

16. The content of the exercise therapy sessions is varied.	☐	☐	☐	☐	☐
17. Recommendations, training plans, or ideas from the sports and exercise therapy program help me motivate my child to be active outside the sessions (e.g., on weekends).	☐	☐	☐	☐	☐

Even **outside** the sports and exercise therapy sessions, there are opportunities for your child to be physically active while on the ward. We would now like to know whether you are satisfied with the available opportunities for movement on the ward.

**Questions About Opportunities for Physical Activity on the Ward**					
	Agree	Some-what agree	Neutral	Some-what disa-gree	Disa-gree
18. I am satisfied with the opportunities for my child to be physically active during inpatient stays (e.g., exercise equipment, table tennis, Table Football).	☐	☐	☐	☐	☐
19. I would like more opportunities for my child to be physically active during inpatient stays.	☐	☐	☐	☐	☐
20. It would be easier for my child to be active during inpatient stays if more equipment or materials were available.	☐	☐	☐	☐	☐
21. I would like more guidance on how to motivate my child to be more active during hospital stays.	☐	☐	☐	☐	☐
22. I would like opportunities for physical activity for myself during my child's inpatient stays.	☐	☐	☐	☐	☐

23. We would like to know if there is anything that could be improved in the sports and exercise therapy program during **inpatient stays**. Suggestions are welcomed here:

____
____
____
____
____
____
____
____

ID										
Date			.			.				

Between hospital stays, your child spends periods of time at home. During this time, there is usually only limited contact with an exercise therapist. This section focuses on the sports and exercise program during **outpatient/outpatient phases** of your child's treatment.

**Questions About the Physical Activity Program**					
	Agree	Some-what agree	Neutral	Some-what disa-gree	Disa-gree
24. I would like more contact with an exercise thera-pist during my child's outpatient phases.	☐	☐	☐	☐	☐
25. I would like more opportunities for my child to be physically active during outpatient visits to the day clinic/ambulatory care.	☐	☐	☐	☐	☐
26. I would like more recommendations, training plans, or ideas to make my child's daily life more active during outpatient phases.	☐	☐	☐	☐	☐
27. I would like more supervised exercise therapy ses-sions for my child during non-inpatient/outpatient phases.	☐	☐	☐	☐	☐
28. Can you imagine your child participating in an online training program during outpatient phases?	☐	☐	☐	☐	☐
29. Can you imagine your child enjoying an online training program?	☐	☐	☐	☐	☐
30. We have the necessary technical equipment (lap-top, tablet, internet access) to participate in an online training program.	☐	☐	☐	☐	☐
31. My child would have time to participate in an online training program during outpatient phases.	☐	☐	☐	☐	☐

What type of physical activity program would your child be interested in participating in during **outpatient phases**? Suggestions are welcomed here:

____

____

____

____

____

____

____

____

Thank you very much! ☺
