# Supplementary material for: Status Quo analysis of an exercise therapy care model in pediatric oncology during acute therapy: perspectives from patients, parents, siblings, and staff
Source: Front Pediatr. 2026 Apr 22;14:1791439. doi: 10.3389/fped.2026.1791439 (PMC13144044; doi:10.3389/fped.2026.1791439)
Supplement: Supplementary file 4 [file Datasheet4.pdf]

Translated Version

Siblings – Acute Therapy
From 6 years

<u>Contact</u>

Exercise Scientist
Pediatric Oncology and Hematology
Oncological Movement Medicine Group
Phone +49 221 478-42646
Email lena.boehlke@uk-koeln.de

Dear ___________________________________,

As part of the

Status Quo Analysis of the exercise project
Department of Pediatric Oncology at the University Hospital of Cologne

we are conducting a survey on the provision of sports and exercise therapy services in pediatric oncology at University Hospital Cologne. Since December 2020, exercise therapy has been offered in addition to the existing treatment services. The purpose of this survey is to identify potential barriers that may limit access to exercise therapy. Our goal is to sustainably improve the structure of exercise therapy and to adapt it to the individual needs and preferences of patients.

Note on completing the questionnaire:

- If you are still too young to fill out, your parents can of course help you
- There are no "right" or "wrong" answers
- If a question applies less to you or you find it difficult to decide on an answer, please tick the answer that spontaneously applies most to you.
- Please mark the answer that applies to you with a cross

Thank you very much, you help us a lot with this!

ID										
Date			.			.				

ID										
Date			.			.				

Who fills out the questionnaire?

- ☐ Child / adolescent alone
- ☐ Child / adolescent with caregiver
- ☐ Parent / caregiver alone

At the beginning, we would like to know some general information about you.

**Personal data**			
Name			
Forename			
Zip Code, City			
Date of birth	___________.______________<br>Month Year		
Place of birth			
Nationality			
Sex	☐ female	☐ male	☐ miscellaneous
Do you go to kindergarten?	☐ yes<br>☐ no		
School	☐ Elementary school<br>☐ Secondary school<br>☐ Secondary school<br>☐ Comprehensive school	☐ Grammar school<br>☐ Special school<br>☐ In training<br>☐ Study	
Number of siblings	☐ 0<br>☐ 1	☐ 2<br>☐ 3	☐ 4<br>☐ More than 4
How long ago was your sibling diagnosed?	☐ between 2 and 6 months<br>☐ more than 6 months		

ID										
Date			.			.				

Your brother / sister has been undergoing anticancer treatment for some time and has become acquainted with the exercise therapy program. We would also like to ask you a few questions about the exercise therapy program.

**General questions about exercise therapy**					
	Agree	Some-what agree	Neutral	Some-what disa-gree	Disa-gree
	😊	🙂	😐	🙁	☹
1. I got to know the exercise program on the ward.	☐	☐	☐	☐	☐
2. I know who to contact if I have questions about exercise and/or sport during my sibling's therapy.	☐	☐	☐	☐	☐
3. I am well aware of the importance of physical activity during my sibling's therapy.	☐	☐	☐	☐	☐
4. The exercise program helps me and my brother / sister to be active.	☐	☐	☐	☐	☐
5. I would like to take advantage of the exercise therapy program together with my sibling more often.	☐	☐	☐	☐	☐
6. Recommendations/ training-plans /ideas of exercise therapy help me to move together with my sibling.	☐	☐	☐	☐	☐
7. I would like more support in moving together with my sibling.	☐	☐	☐	☐	☐

8. We would like to know if there is anything we can improve in the exercise therapy program for you. **Suggestions are welcomed here:**

____
____
____
____
____
____
____
____

ID										
Date			.			.				

Now we would like to learn more about what obstacles there are for you to exercise together with your sibling.

**Obstacles**					
	Agree	Some-what agree	Neutral	Some-what disa-gree	Disa-gree
9. I'm afraid to exercise with my sibling.	☐	☐	☐	☐	☐
10. I am afraid that my sibling could overexert himself or injure himself.	☐	☐	☐	☐	☐
11. My sibling feels too weak to exercise with me.	☐	☐	☐	☐	☐
12. We would like to exercise together, but we don't know how.	☐	☐	☐	☐	☐
13. We prefer to deal with other things together.	☐	☐	☐	☐	☐

Now we would like to learn more about the reasons why you exercise or do sports together with your sibling.

**Motives**					
	Agree	Some-what agree	Neutral	Some-what disa-gree	Disa-gree
I move or do sports together with my sibling, ...					
14. ... to sleep better.	☐	☐	☐	☐	☐
15. ... to strengthen my sibling.	☐	☐	☐	☐	☐
16. ... to spend time together with my sibling.	☐	☐	☐	☐	☐
17. ... to do something good for my sibling.	☐	☐	☐	☐	☐
18. ... to forget the illness for a moment.	☐	☐	☐	☐	☐
19. ... to have fun and joy.	☐	☐	☐	☐	☐
20. ... Because others want us to move.	☐	☐	☐	☐	☐

Thank you very much! ☺
