# Supplementary material for: Status Quo analysis of an exercise therapy care model in pediatric oncology during acute therapy: perspectives from patients, parents, siblings, and staff
Source: Front Pediatr. 2026 Apr 22;14:1791439. doi: 10.3389/fped.2026.1791439 (PMC13144044; doi:10.3389/fped.2026.1791439)
Supplement: Supplementary file 5 [file Datasheet5.pdf]

Staff - Acute Therapy

Contact

Exercise Scientist
Pediatric Oncology and Hematology
Oncological Movement Medicine Group
Phone +49 221 478-42646
Email lena.boehlke@uk-koeln.de

As part of the

Status Quo Analysis of the exercise therapy project of the Department of Pediatric Oncology at the University Hospital of Cologne

we are conducting a survey on the provision of sports and exercise therapy services in pediatric oncology at University Hospital Cologne. Since December 2020, exercise therapy has been offered in addition to the existing treatment services. The purpose of this survey is to identify potential barriers that may limit access to exercise therapy. Our goal is to sustainably improve the structure of exercise therapy and to adapt it to the individual needs and preferences of patients.

Note on completing the questionnaire:

- If a question applies less to you or you find it difficult to decide on an answer, please tick the answer that spontaneously applies most to you.
- Please mark the answer that applies to you with a cross.
- The questionnaire is evaluated anonymously, so your information on the questionnaire cannot be associated with your name. The information is used exclusively for the evaluation of the exercise therapy program.

Thank you for taking the time!

ID										
Date			.			.				

ID										
Date			.			.				

Information about yourself and your work at the University Hospital Cologne

My work in the pediatric oncology department of the University Hospital of Cologne	☐ Nursing staff<br>☐ Doctor<br>☐ Psychosocial care (e.g. social pedagogue, psychologist, remedial teacher, etc.)<br>☐ Sports, exercise or physiotherapist<br>☐ Other
How long have you been working in the paediatric oncology department at the University Hospital of Cologne?	☐ less than 1 year<br>☐ between 1 and 5 years old<br>☐ between 5 and 10 years old<br>☐ more than 10 years

This section is about the sports and exercise therapy care of patients **during anticancer treatment.**

**Questions about the exercise therapy program**						
	Agree	Somewhat agree	Neutral	Somewhat disagree	Disagree	I can't judge
1. I feel sufficiently informed about the relevance of physical activity during anticancer therapy.	☐	☐	☐	☐	☐	☐
2. I feel sufficiently informed about the exercise therapy program.	☐	☐	☐	☐	☐	☐
3. I consider the exchange with the exercise therapist to be important and helpful.	☐	☐	☐	☐	☐	☐
4. I consider the exchange with the exercise therapist to be sufficient.	☐	☐	☐	☐	☐	☐
5. The exercise therapists are easy to reach if I have a concern.	☐	☐	☐	☐	☐	☐
6. I would like to see more exchange with the exercise therapists.	☐	☐	☐	☐	☐	☐
7. In my work, I motivate patients to take advantage of the exercise therapy during their anticancer therapy.	☐	☐	☐	☐	☐	☐
8. In my work, I motivate parents / guardians to motivate their children to exercise therapy during anticancer therapy.	☐	☐	☐	☐	☐	☐

ID										
Date			.			.				

9. I have the feeling that patients **receive sufficient exercise therapy** during their inpatient stays.	☐	☐	☐	☐	☐	☐
10. I have the feeling that patients **receive sufficient exercise therapy** care during their outpatient stays.	☐	☐	☐	☐	☐	☐
11. I have the feeling that parents are sufficiently informed about the relevance of physical activity during anticancer therapy.	☐	☐	☐	☐	☐	☐
12. I find the exercise program during my work disturbing.	☐	☐	☐	☐	☐	☐
13. I have the feeling that routine processes are disturbed by the exercise program.	☐	☐	☐	☐	☐	☐
14. I have the feeling that the exercise program supports my work with/on patients.	☐	☐	☐	☐	☐	☐
15. I have the feeling that the exercise program supports my work with the parents/guardians.	☐	☐	☐	☐	☐	☐

**Here you are welcome to post suggestions, wishes and suggestions for improvement:**
____
____
____
____
____
____

Thank you very much! ☺
