# Supplementary figures and images for: Status Quo analysis of an exercise therapy care model in pediatric oncology during acute therapy: perspectives from patients, parents, siblings, and staff
Source: Front Pediatr. 2026 Apr 22;14:1791439. doi: 10.3389/fped.2026.1791439 (PMC13144044; doi:10.3389/fped.2026.1791439)

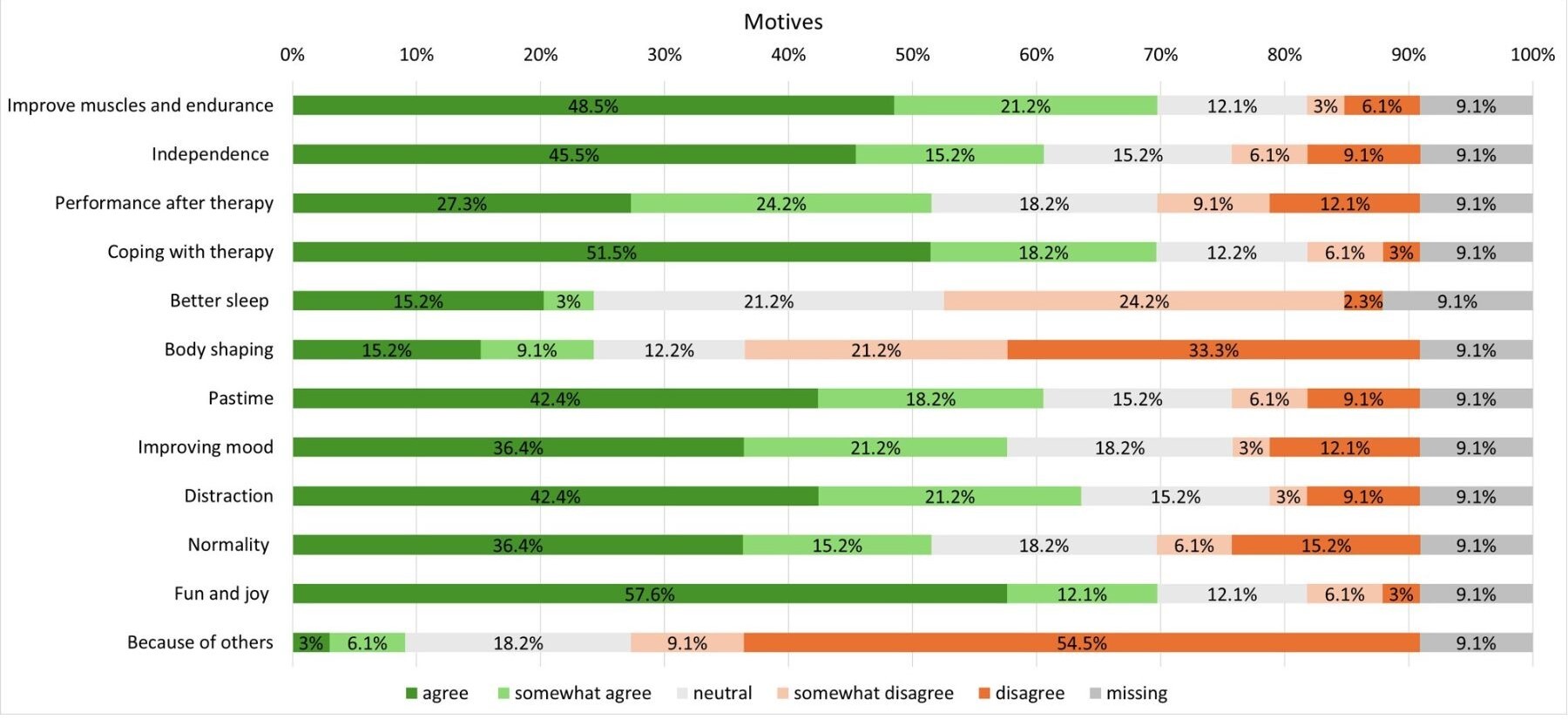

Supplement: Supplementary file 6 [file Image1.jpeg]

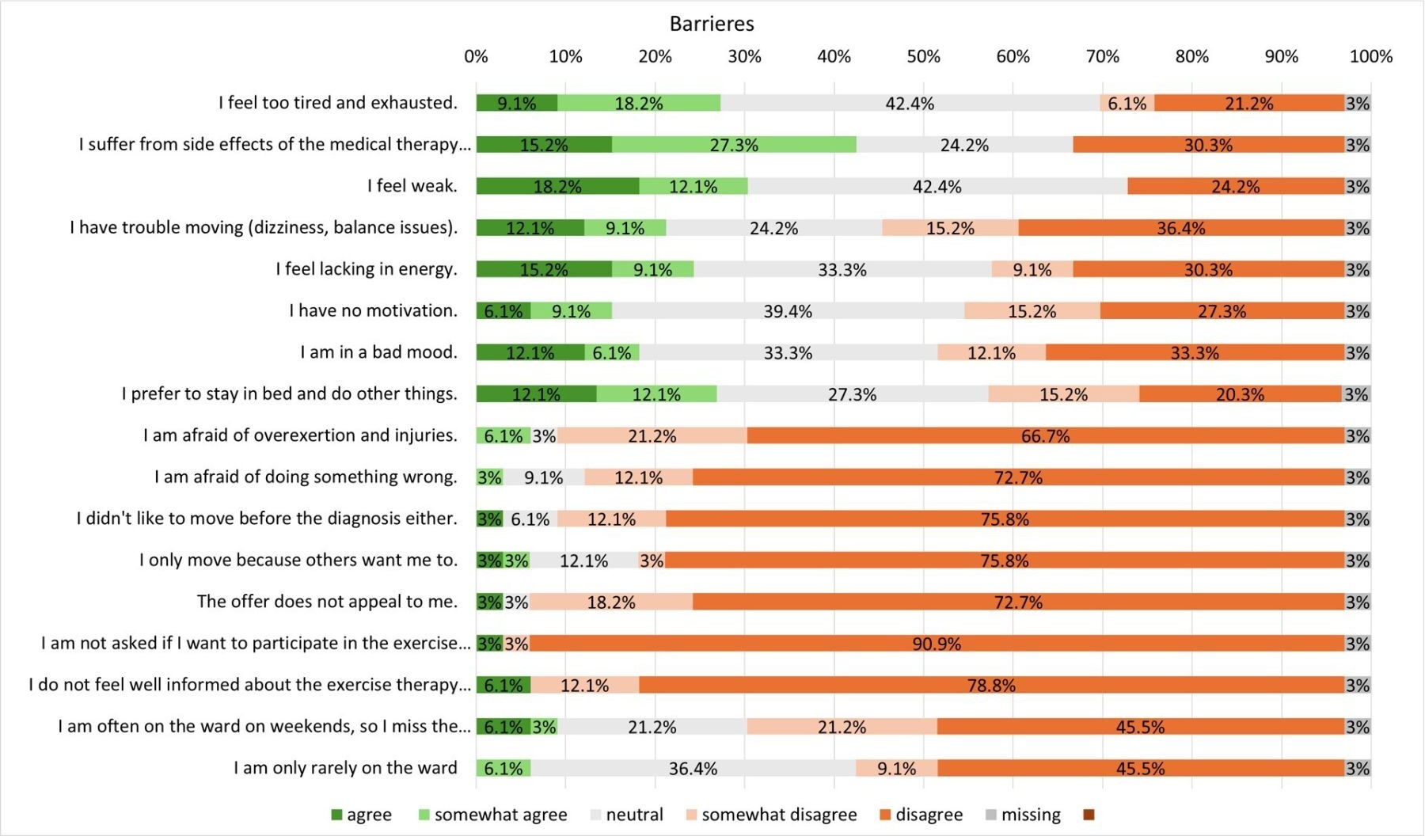

Supplement: Supplementary file 7 [file Image2.jpeg]

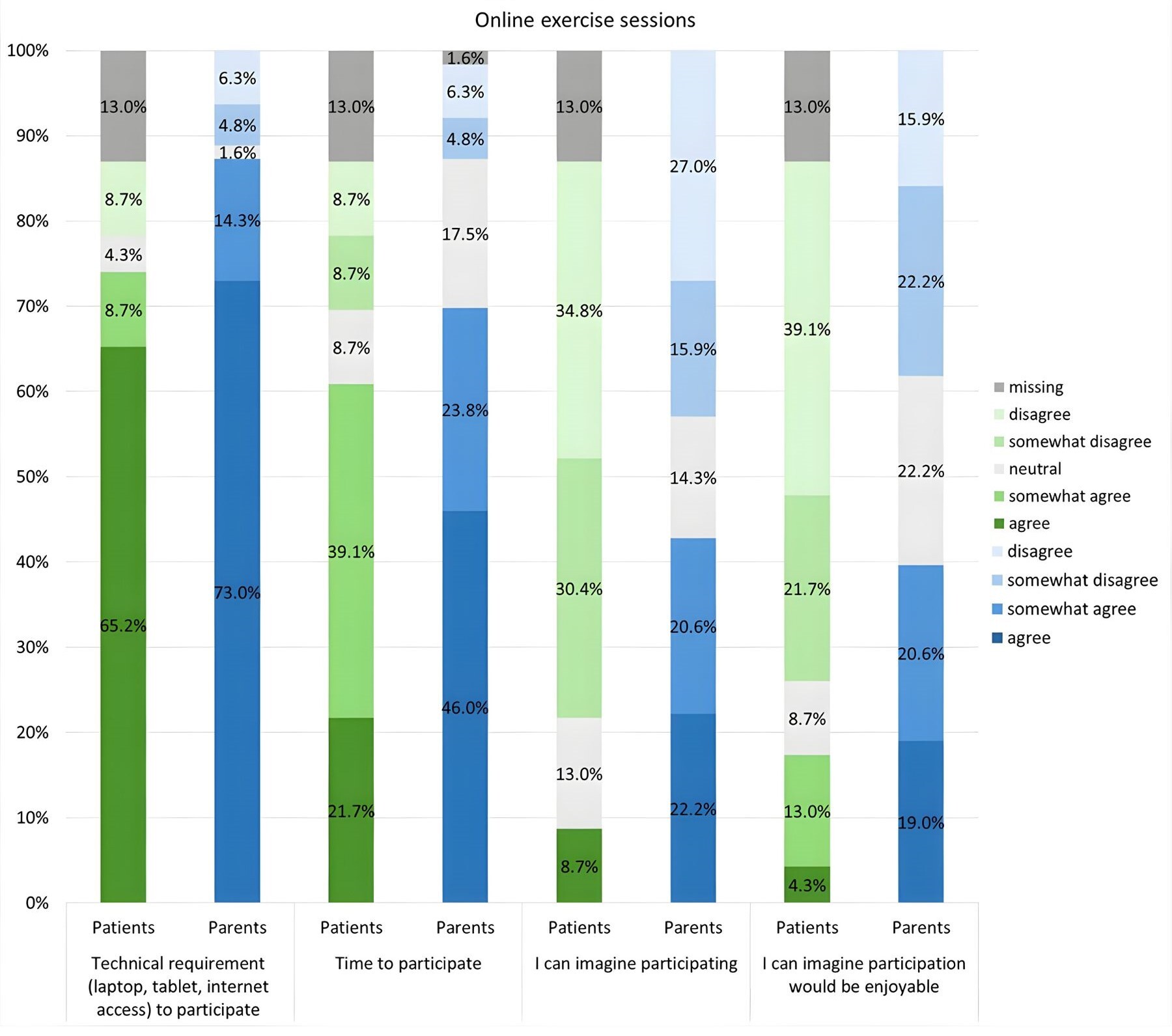

Supplement: Supplementary file 8 [file Image3.jpeg]

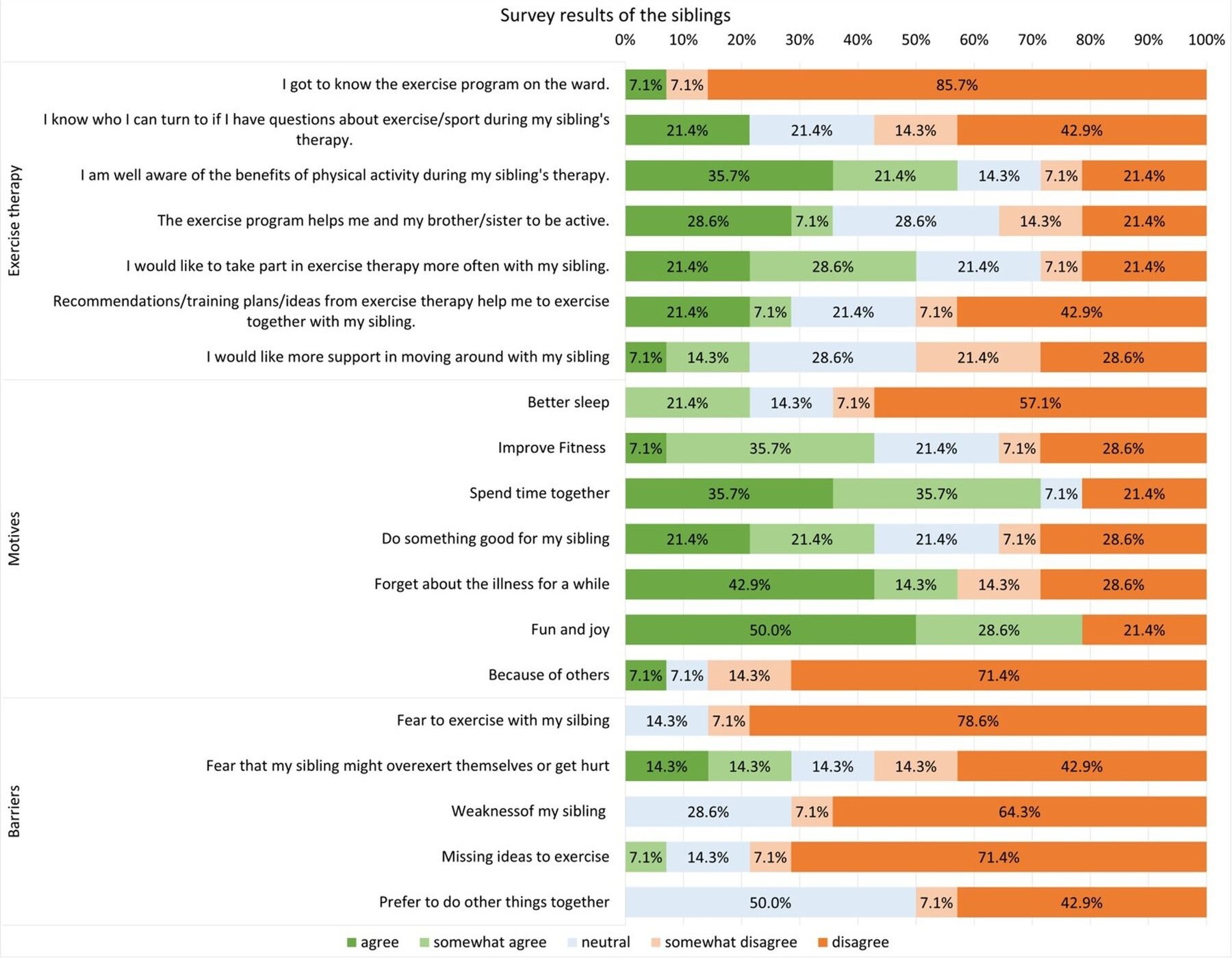

Supplement: Supplementary file 9 [file Image4.jpeg]
